# Age-Related Pathology in Corticobasal Degeneration

**DOI:** 10.3390/ijms25052740

**Published:** 2024-02-27

**Authors:** Maya Mimuro, Yasushi Iwasaki

**Affiliations:** 1 Department of Pathology, Mie University Hospital, Tsu 514-8507, Japan; 2 Institute for Medical Science of Aging, Aichi Medical University, Nagakute 480-1195, Japan; iwasaki@sc4.so-net.ne.jp

**Keywords:** corticobasal degeneration, tau, aging, Alzheimer’s disease, ARTAG, TDP-43, LATE, α-synuclein, amygdala-predominant Lewy body disease

## Abstract

Elderly human brains are vulnerable to multiple proteinopathies, although each protein has a different transmission pathway. Tau-immunoreactive astrocytes are well-known in elderly brains. In contrast, astrocytic plaques, a hallmark in corticobasal degeneration (CBD), rarely occur in aging and neurodegenerative disease other than CBD. To elucidate the clinicopathological correlation of aging-related pathology in CBD, we examined 21 pathologically proven CBD cases in our institute (12 males and 9 females, with a mean age of death 70.6 years). All CBD cases showed grains and neurofibrillary tangles (NFTs). Fifteen cases (71.4%) showed beta-amyloid deposition such as senile plaques or cerebral amyloid angiopathy. Three cases (14.3%) had Lewy body pathology. One case was classified as amygdala-predominant Lewy body disease, although no cases met the pathological criteria for Alzheimer’s disease. Five cases (23.8%) displayed Limbic-predominant and age-related TDP-43 encephalopathy (LATE). NFTs, grains, and TDP-43-positive neuronal inclusions were widely distributed throughout the limbic system of CBD patients, but their densities were low. CBD might a have similar cell vulnerability and transmission pathway to that of multiple proteinopathy in aging brains.

## 1. Introduction

Brains in elderly patients are known to have comorbid pathologies such as neurodegenerative diseases and cerebrovascular disorders [1,2]. Aging brains have different types of abnormal protein accumulations. For example, the brains of cases with Alzheimer’s disease (AD), a common disease in elderly patients, revealed two different abnormal protein accumulations: tau and β-amyloid. In the aging brain, Lewy body disease (LBD), one of the α-synucleinopathies, is more common. The hippocampus and medial temporal lobe are the most affected regions in aging and AD. The hippocampus is well-known as a center of memory, and memory loss is the most prominent symptom of AD patients [3]. In LBD, α-synuclein deposits are commonly found in the amygdala. The amygdala is closely linked to emotion. The limbic system, including the hippocampus and the amygdala, is easily impaired in aging and neurodegenerative diseases. Recently, two new entities associated with age-related pathology have been reported: aging-related tau astrogliopathy (ARTAG) [4] and limbic age-related TDP-43 encephalopathy (LATE) [5]. ARTAG is an astrocytic tau pathology observed in the subpial and subependymal regions of the gray matter and the white matter of the aging brain. LATE is defined by a stereotypical TDP-43 proteinopathy, especially in the limbic region of the aging brain. Also, age-related pathology, including AD and LBD, plays an important role in improving the clinical diagnosis and prognosis of neurodegenerative diseases. Argyrophilic grain disease (AGD) is an age-related, four-repeat tauopathy characterized by spindle- or comma-shaped argyrophilic grains [6,7]. Interestingly, several researchers have reported that AGD incidence is much higher with progressive supranuclear palsy (PSP) and corticobasal degeneration (CBD) than with AD or in controls [8,9,10]. PSP and CBD are two major diseases of four-repeat tauopathies. Pathologically, both diseases have four-repeat tau inclusions in the glia and neurons, mainly in the basal ganglia, the substantia nigra, and the frontal lobe or the parietal lobe, not the limbic system. AGD also has four-repeat tau inclusions, although tau-positive lesions are mainly in the neuronal dendrites and axons. Tufted astrocytes are the neuropathological hallmark of PSP [11,12]. However, the existence of a small number of tufted astrocytes has been reported in the basal ganglia or brainstem nuclei in AGD [13,14], although they do not fulfill the pathological criteria of PSP [11]. Astroglial tau pathologies are currently emphasized. In ARTAG, thorn-shaped astrocytes (TSAs) show a characteristic fibrillar and short-branched morphology containing four-repeat tau [4]. TSAs are frequent in the aging brain and in the brain of individuals affected by tauopathies including PSP [15,16,17]. At the point of four-repeat tau pathology, PSP appears to be closely related to AGD or the age-related pathology.

Despite the higher frequency of AGD in CBD than in PSP, astrocytic plaques, neuropathological hallmarks of CBD [18,19,20], are rare to find in brains with other neurodegenerative disease, including tauopathies or the aging brain [14]. However, swollen achromatic neurons, commonly called ballooned neurons, are neuropathological hallmarks of both CBD [21] and AGD [22]. Ballooned neurons may also occur in limbic lesions in AD [23]. CBD patients exhibit a variety of clinical manifestations, including dementia similar to frontotemporal dementia (FTD) or AD [24]. Is CBD related to aging pathology? CBD has a much lower prevalence than PSP, so it is difficult to study using many CBD brains. CBD has neuropathologically abundant argyrophilic threads, glial tau-immunoreactive thread-like processes, in the affected areas of gray matter and white matter [25]. Therefore, the evaluation of TDP-43-positive inclusions and other tau-positive inclusions, like TSAs or neuropil threads, is challenging. The relationship between CBD and the age-related pathology is still unclear.

We had a chance to evaluate 21 CBD brains and assessed the age-related pathology, including a new entity: LATE. The amygdala and the hippocampus regions were obtained from paraffin-embedded samples; stained with Gallyas–Braak stains [26]; and subjected to immunohistochemistry using anti-phosphorylated α-synuclein, anti-β-amyloid, anti-phosphorylated tau (AT8), anti-pan TDP-43, anti-phosphorylated TDP-43, anti-human 3-repeat tau (RD3), and anti-human 4-repeat tau (RD4). As mentioned in the Materials and Methods section, we evaluated the age-related pathology using established criteria.

## 2. Case Presentation

### 2.1. Results

#### 2.1.1. Clinical Phenotypes

Twenty-one cases were classified as four phenotypes based on the clinical criteria for CBD: clinical corticobasal syndrome (CBS), frontal behavioral–spatial syndrome (FBS), nonfluent/agrammatic variant of progressive aphasia (naPPA), and progressive supranuclear palsy syndrome (PSPS), in addition to other types that were not classified as any of the four clinical syndromes [27]. CBS exhibits an asymmetric presentation of levodopa-resistant parkinsonism, dystonia, myoclonus, orobuccal or limb apraxia, and alien limb phenomena. In our study, four cases were classified as CBS. FBS shows executive dysfunction, behavior or personality changes, and visuospatial deficits. Two cases were of the FBS type. PSPS has similar symptoms to PSP, for example, axial or symmetric limb rigidity or akinesia, postural instability or falling, urinary incontinence, behavioral changes, supranuclear vertical gaze palsy, decreased velocity, or vertical saccades. 10 cases were classified as PSPS, and 4 cases were classified as other types. naPPA is characterized by aphasia, e.g., effortful agrammatic speech, impaired grammar comprehension with relatively preserved single-word comprehension, or grouping and distorted speech production. Only one case fulfilled naPPA criteria (Table 1).

#### 2.1.2. Pathological Phenotypes

All our cases fulfilled the neuropathologic criteria for CBD, including astrocytic plaque, argyrophilic threads, and ballooned neurons [20]. Tau-positive lining was also observed in the subpial or subependymal locations of all cases. The pathological features of our cases were divided into three representative subtypes as we proposed before [28,29]: 12 cases with typical CBD type, 8 cases with PSP-like type, and only 1 case with basal ganglia-predominant type (Table 1).

#### 2.1.3. Argyrophilic Grain Disease

All 21 cases had argyrophilic grains with pre-tangles and ballooned neurons (Table 1): 10 cases with Saito’s AGD stage I, 8 cases with Saito’s AGD stage II, and 3 cases with Saito’s AGD stage III. The distribution was wide in all cases, but the density was low. CBD cases were difficult to classify directly using Saito’s AGD stage. Therefore, we assessed grains according to the density of Saito’s AGD stage.

#### 2.1.4. Alzheimer Disease-Related Neurofibrillary Pathology and Amyloid β (Aβ)-Plaque Pathology

All 21 cases showed three-repeat tau-positive tau pathology (Table 1): seven cases with Braak’s NFT stage I, nine cases with Braak’s NFT stage II, three cases with Braak’s NFT stage III, and one case with Braak’s NFT stage IV. In most cases, the NFT density was low, but the distribution was relatively wide. None had a Braak’s NFT stage greater than IV. Furthermore, neuropil threads related to AD were scarce in most cases. However, they had far more glial threads related to CBD than neuropil threads. The case with Braak’s NFT stage IV was clinically classified as frontal behavioral–spatial syndrome and had severe brain atrophy.

A total of 15 of 21 cases showed Aβ deposition in some area of the brain (Table 1): 12 cases with senile plaques, 6 cases with Thal’s amyloid phase 1, 4 cases with Thal’s amyloid phase 2, 2 case with Thal’s amyloid phase 3, and 1 case with Thal’s amyloid phase 4. A total of 9 of 21 cases had no diffuse plaques or neuritic plaques. Neuritic plaque frequency was low. Only five cases had neuritic plaques: two cases with CERAD a neuritic plaque score of “Sparse” and three cases with a CERAD neuritic plaque score of “Moderate”. Four of nine cases with senile plaques had only diffuse plaques.

A total of 9 of 21 cases had cerebral amyloid angiopathies (CAA) (Table 1): 7 cases with a Love’s hybrid protocol score of 1 and two cases with a Love’s hybrid protocol score of 2. The presence of CAA alone without a senile plaque was seen in two out of nine cases.

#### 2.1.5. Lewy Body Pathology

**Figure 1:**
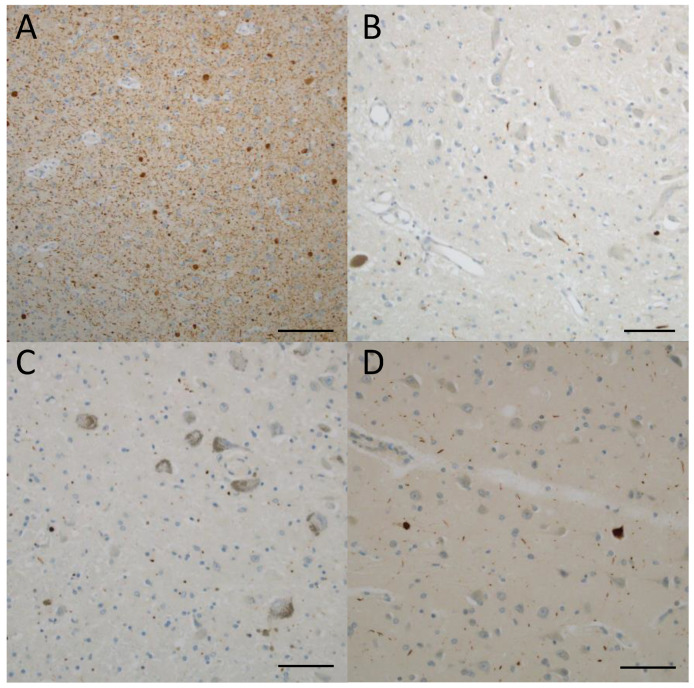
Lewy pathology. (**A**–**D**) Case 12 (clinical phenotype, unclassified; pathological phenotype, typical). The amygdala (**A**) shows many Lewy bodies and Lewy neurites. A few Lewy dots are shown in the dorsal motor nucleus of the vagal nerve (**B**) and the locus coeruleus (**C**). Several Lewy bodies and Lewy neurites are shown in the cingulate gyrus (**D**). (**A**–**D**) phosphorylated alfa synuclein. Scale bars: (**A**) 100 μm; (**B**–**D**) 50 μm.

Among 21 cases, 3 showed α-synuclein deposition in some part of the brain (Table 1). One of them was brainstem-predominant according to the classification of the Lewy body pathology fourth consensus report of the DLB consortium, one was limbic, and one was amygdala-predominant. In this case, the amygdala showed severe synuclein pathology, especially Lewy neurites (Figure 1A). Ballooned neurons and argyrophilic threads co-exist with Lewy pathology. A-synuclein-positive small dots were found in the dorsal motor nucleus of the vagal nerve and the locus coeruleus in the amygdala-predominant case (Figure 1B,C). In addition, there were several Lewy bodies in the cingulate gyrus (Figure 1D). One case showed α-synuclein pathology in the intermediolateral nucleus and sympathetic postganglionic neurons but no Lewy bodies or Lewy dots anywhere in the brain.

#### 2.1.6. Limbic-Predominant and Age-Related TDP-43 Encephalopathy (LATE)

**Figure 2:**
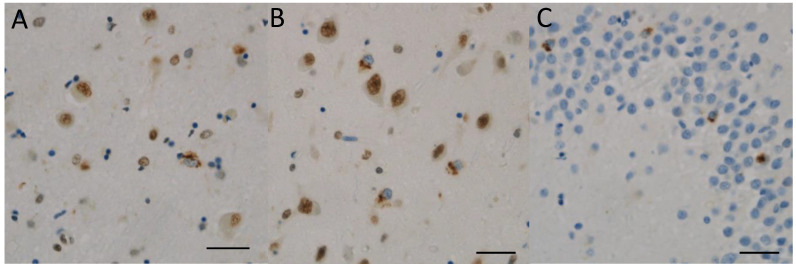
TDP-43 pathology in the limbic system. (**A**,**B**) Case 10 (clinical phenotype, PSPS; pathological phenotype, PSP-like). (**C**) Case 6 (clinical phenotype, PSPS; pathological phenotype, PSP-like). Several TDP-43 positive inclusions are shown in the amygdala (**A**) and the entorhinal cortex (**B**). Their nuclei are negative for TDP-43 immunoactivity. Several TDP-43-positive intracytoplasmic inclusions are shown in the hippocampal granule cells (**C**). (**A**–**C**) TDP-43. Scale bars: (**A**–**C**) 20 μm.

A total of 5 of 21 cases showed TDP-43-positive inclusions in the limbic areas: three with LATE stage 1 and two with LATE stage 2 (Figure 2). The distribution was wide in these CBD cases, but the density was low. It was difficult to directly classify the TDP-43-positive inclusions using the LATE stage in CBD cases. In light of this, we assessed the TDP-43-positive inclusions primarily based on the density of LATE stages according to the consensus working group report for LATE.

### 2.2. Materials and Methods

#### 2.2.1. Subjects

Twenty cases derived from Aichi Medical University Karei Ikagaku Brain Resource Center (AKBRC) between 1994 and 2020 were enrolled in this study (Table 1). All cases fulfilled pathological criteria for CBD diagnosis [20]. Twelve cases were male, and nine were female. The average age at disease onset was 63.5 years (ranging from 53 to 81 years), the average age at death was 70.6 years (ranging from 60 to 86 years), and the average duration of the disease was 7.1 years (ranging from 3 to 11 years). The present study excluded cases with positive family histories of corticobasal syndrome (CBS). Clinical features of all patients were retrospectively reviewed from their medical records.

#### 2.2.2. Tissue Preparation and Immunohistochemical Procedures

The brains were fixed in 20% buffered formalin and cut into coronal sections. Blocks were embedded in paraffin and cut into 4.5 or 9 μm-thick sections. The regions are systematic in our institute as follows: the isocortex of frontal, temporal, and parietal cortices; subcortical white matter of the frontal and temporal lobes; anterior cingulate gyrus; insular cortex; primary motor cortex; amygdala; hippocampus; basal ganglia; midbrain; pons; medulla oblongata; cerebellum; and spinal cord. For routine histological examination, all sections were stained with HE and KB. To assess the neuronal and glial inclusions, we selected several sections and stained them with the modified Gallyas–Braak method (GB) and several immunohistochemical stains [9,26]. The following primary antibodies were used: anti-alpha-synuclein (rabbit polyclonal, 1:1000, Sigma Aldrich, St. Louis, MO, USA), anti-phosphorylated alfa-synuclein (pSyn#64, mouse monoclonal, 1:1000, Wako Pure Chemical Industries, Osaka, Japan), anti-beta-amyloid (clone 6F/3D, mouse monoclonal, 1:100, Dako, Glostrup, Denmark), anti-phosphorylated tau (clone AT-8, mouse monoclonal, 1:1000, Innogenetics, Ghent, Belgium), anti-pan transactivation response DNA-binding protein 43 kDa (TDP-43) (rabbit polyclonal, 1:2000; Proteintech, Chicago, IL, USA), anti-phosphorylated TDP-43 (pTDP-43 s409/410, rabbit polyclonal, 1:3000; Cosmobio, Tokyo, Japan), anti-3 repeat-tau (RD3, mouse monoclonal, 1:500, EMD-Millipore, Burlington, MA, USA), and anti-4-repeat-tau (RD4, mouse monoclonal, 1:500, EMD-Millipore). As a secondary detection system, the standard avidin/biotin method was performed. Diaminobenzidine was used as a chromogen. All immunostained sections were lightly counterstained with Mayer’s hematoxylin.

#### 2.2.3. Neuropathological Assessment

All cases were pathologically proven as CBD using established criteria [20]: characteristic tau-immunoreactive neuronal and glial lesions (in particular, astrocytic plaques and thread-like processes in the gray and white matter, in focal cortical lesions, and in the substantia nigra) and ballooned neurons in affected cortices.

Aging-related pathology was assessed in accordance with established criteria: Braak’s criteria [30] for neurofibrillary tangles (NFTs) and Thal’s criteria [31] and Consortium to Establish a Registry for Alzheimer’s Disease (CERAD) plaque scores [32] for senile plaques. We scored argyrophilic grains based on Saito’s stage [33]: stage I, abundant grains spread into the parahippocampal area; stage II, abundant grains spread into the occipitotemporal gyrus; stage III, abundant grains spread into the inferior aspect of the inferior temporal gyrus. We used the fourth consensus report of the DLB consortium [34] for Lewy body disease: brainstem-predominant, Lewy bodies mainly spread into brainstem regions; limbic (transitional), Lewy bodies mainly spread into limbic regions; diffuse neocortical, Lewy bodies spread into neocortical regions; amygdala-predominant, Lewy bodies mainly spread into the amygdala, with few Lewy bodies in brainstem regions. We scored TDP-43 neuronal cytoplasmic inclusions (NCIs) in the limbic system based on the consensus working group report for LATE: grade 0, none; grade 1, NCIs spread into the amygdala; grade 2, NCIs spread into the hippocampus; grade 3, NCIs spread into the middle frontal gyrus in the prefrontal area. We used Love’s hybrid protocol [35] to score cerebral amyloid angiopathy (CAA) and CAA-associated vasculopathy; score 0, CAA is absent; score 1, scant β-amyloid deposition at parenchymal and meningeal CAA; score 2, some circumferential β-amyloid deposition at parenchymal and meningeal CAA; score 3, widespread circumferential β-amyloid deposition at parenchymal and meningeal CAA. We assessed NFT staging using AT-8, RD3, and RD4 immunohistochemistry staining. The judgement was undertaken by two researchers (M.M. and Y.I.) blinded to case identification.

## 3. Discussion

Among pathologically confirmed CBD cases, 37.1% had the CBS phenotype, and 23.3% had PSPS [27]. In this study, around half of 21 cases were classified as PSPS (10 cases, 47.6%), and 4 cases were classified as CBS (19.0%). The reason may be that the number of investigated cases was small and that atypical cases are more likely to be encountered in an autopsy. On the other hand, there was a high correlation between the clinical phenotype and the pathological phenotype of each CBD case. This was based on the fact that CBD diagnostic accuracy has improved as neurologists have gained a great deal of experience in diagnosing patients with CBD.

Pathologically, our CBD cases showed a low incidence of Alzheimer-related pathology. No case showed “intermediate” or “high” AD neuropathologic change as evaluated by ABC score for AD neuropathologic change [32]. Aging brains have the highest incidence of neurodegenerative proteinopathies: Alzheimer-related pathology is seen 19–100%, Lewy pathology in 6–39%, TDP-43 proteinopathy in 6–39%, and mixed pathologies in 10–93% [1,36,37]. Alzheimer’s-related pathology and Lewy body pathology are suggested to be prevalent in elderly people with or without cognitive dysfunction. Considering the average age of death was 70.6 ± 7.0 years old, the frequency of Alzheimer’s-related pathology in our CBD brains is much lower than in aging brains.

An entity called LATE has recently been characterized by a stereotypical TDP-43 proteinopathy in older adults, particularly those with Alzheimer-related pathology [5,38]. CBD, PSP, and other neurodegenerative diseases have been known to occur less frequently in elderly brains [1,2,37]. In our CBD cases, the incidence of TDP-43 proteinopathy was 23.8% (5 cases). In contrast, we stand on the side of four-repeat tauopathy, with AGD occurring more frequently in PSP and CBD than in AD or in cases with cognitive dysfunction [8,9,10]. All our CBD cases cooccurred with Saito’s stage I to III AGD. In 2016, the ARTAG evaluation strategy was announced [4]. In the report, tau-immunoreactive astrocytic cytopathologies are classified into two pathologies: primary tauopathy-related astroglial tau pathology and ARTAG-related astroglial tau pathology. Tufted astrocytes, astrocytic plaques, globular astroglial inclusions, and ramified astrocytes are present in primary tauopathy-related astroglial tau pathology. Each astrocytic inclusion characterizes each specific disease; for example, tufted astrocytes are characteristic of PSP, and astrocytic plaques are a hallmark of CBD. ARTAG-related astroglial tau pathology is literally related to aging brains. TSAs and granular or fuzzy astrocytes (GFAs) are classified in this group. In CBD brains, the TSA frequency is high [4,15,17,39]. All our cases had tau-positive lining in the subpial or subependymal locations. Several studies have reported TSA in the gray matter or white matter of CBD brains [4,15,17,39], but in most of our cases, TSA was difficult to distinguish from argyrophilic threads. One of the characteristic pathologies of CBD is subpial astroglial tau pathology [40]. Moreover, several researchers speculate that neuronal pathology is abundant in end-stage disease and gradually overtakes astroglial tau pathology in CBD or PSP [41]. Therefore, we excluded ARTAG assessment from this study. Compared with AGD or AD cases, grain density, NFT density, and TDP-43-positive neuronal inclusion density in our CBD cases were sparse, with wide distributions. These features were similar to Kii ALS/PDC cases [42] or centenarians [43,44].

Moreover, one of our CBD cases showed Amygdala-predominant Lewy pathology. The distribution of Lewy pathology seems to follow two overarching patterns: a caudo-rostral pattern that starts in the olfactory bulb and enteric nervous system, extending to the central nervous system (CNS) through the brainstem, and an amygdala-centered pattern that starts in the amygdala, extending to the CNS [45]. According to a generally accepted concept, Lewy pathology follows a hierarchical caudo-rostral progression [46]. Among cases aged over 85 years, 32% showed amygdala-predominant progression. The amygdala-predominant patterns were significantly associated with brains with severe Braak’s NFT stage, high CERAD neuritic plaque score, and APOE e4. However, our case with amygdala-predominant Lewy pathology was 70 years old. His brain had a mild Braak’s NFT stage and a moderate CERAD neuritic plaque score.

Our previous and present results show: (1) NFTs, grains, and TDP-43-positive inclusions were distributed widely; (2) the densities of these inclusions were relatively low; (3) the CBD cases were relatively young; and (4) these inclusions existed with characteristic CBD pathology, like argyrophilic threads or ballooned neurons. These findings suggest that CBD does not have an AD pathology, but our CBD brains had age-related pathologies like AGD, LATE, and amygdala-predominant Lewy pathology. CBD might have similar cell vulnerability and transmission pathway to those of multiple proteinopathy in aging brains. However, our sample size was limited, and “autopsy cases” themselves may have some selection bias, for example, having unusual clinical symptoms. The relationship between CBD and age-related pathology needs to be explored by collecting more CBD cases.

## 4. Conclusions

In conclusion, this study revealed the aging pathology of 21 autopsy-proven CBD cases. None of the CBD cases fulfill the Alzheimer’s disease criteria. However, all CBD cases were combined with AGD, and one case showed amygdala-predominant Lewy body pathology. CBD may play a similar role to aging brains in terms of cell vulnerability and the transmission pathway to multiple proteinopathies.

## Figures and Tables

**Table 1:** Clinicopathological summary of the cases.

Case	Sex	Age of Death	Clinical Duration, Years	Clinical Phenotype ^a^	Brain Weight	Pathological Phenotype ^b^	Thal’s Amyloid Phase	Braak’s NFT Stage	CERAD Neuritic Plaque Score	Love’s CAA Score	Saito’s AGD Stage	Lewy Body Pathology Classification	LATE Stage
1	M	60	7	PSPS	1145	PSP-like	0	II	0	0	II	0	0
2	M	61	3	CBS	1250	typical	1	I	0	0	I	0	0
3	F	62	8	PSPS	990	PSP-like	1	II	0	1	I	0	0
4	F	62	10	naPPA	870	typical	3	II	0	0	II	0	0
5	F	67	6	FBS	975	typical	0	I	0	0	I	0	0
6	F	67	8	PSPS	985	PSP-like	2	I	B	2	II	0	2
7	M	68	4	PSPS	1350	typical	1	I	0	1	II	0	0
8	M	68	8	PSPS	1200	PSP-like	0	I	0	1	I	Brainstem	1
9	M	68	3	PSPS	1270	BG	0	II	A	0	I	0	1
10	M	69	10	PSPS	1015	PSP-like	0	I	0	0	II	0	1
11	F	70	11	FBS	770	typical	2	IV	A	0	III	0	0
12	M	70	8	unclassified	1135	typical	4	II	B	2	I	Amygdala	0
13	M	70	4	unclassified	1161	PSP-like	0	II	0	0	II	0	0
14	F	71	8	PSPS	1020	PSP-like	2	II	0	1	II	0	0
15	M	73	6	CBS	1200	typical	1	III	0	1	I	0	0
16	F	74	10	PSPS	1000	PSP-like	0	III	0	0	I	0	0
17	M	76	7	unclassified	1120	typical	0	II	0	0	I	Limbic	0
18	M	77	5	PSPS	1190	typical	1	II	0	0	II	0	0
19	F	81	11	CBS	1060	typical	1	I	0	1	III	0	2
20	F	83	6	CBS	955	typical	0	III	0	1	I	0	0
21	M	86	6	unclassified	1025	typical	2	I	B	0	III	0	0

PSPS: progressive supranuclear palsy syndrome; CBS: corticobasal syndrome; naPPA: nonfluent/agrammatic variant of primary progressive aphasia; FBS: frontal behavioral–spatial syndrome; typical: typical CBD type; BG: basal ganglia-predominant type; M: male; F: female; NFT: neurofibrillary tangle; CERAD: the Consortium to Establish a Registry for Alzheimer’s disease; CAA: cerebral amyloid angiopathy; AGD: argyrophilic grain disease; LATE: limbic-predominant age-related TDP-43 encephalopathy. ^a^ Armstrong’s criteria for the diagnosis of cortical degeneration [27]. ^b^ Yoshida’s pathological subtypes of CBD [28].

## Data Availability

The data that support the findings of this study are available from the corresponding author upon reasonable request.
